# Supplementary material for: Learning curve and surgical outcome of robotic assisted colorectal surgery with ERAS program
Source: Sci Rep. 2022 Nov 29;12:20566. doi: 10.1038/s41598-022-24665-w (PMC9709162; doi:10.1038/s41598-022-24665-w)
Supplement: Supplementary file 2 — Supplementary Information 2. [file 41598_2022_24665_MOESM2_ESM.zip › RAL Raw data-Ñ[▒K/091/OP N_ñw╝╨░O▒Kñσ.pdf]

W95 / W95

臺中榮民總醫院
手術室護理記錄

[redacted]

基本資料

- 室別： 開刀房-17室，手術科別： CRS，手術類別： 常規，日期： 2021/06/10
- 進入時間： 1345 ； 麻醉開始時間： 1400 ； Time Out時間： 1443 ； 劃刀時間： 1444 ； 手術結束時間： 1910 送出時間： 1945 ；
- 麻醉方式： GE
  診斷：CA OF RECTUM SP CCRT(6/10-17)
  手術名稱：Robot Assisted Low Anterior Resection
  手術局部用藥：NIL
  術前預防性抗生素：
  - 抗生素名稱一： Veterin 1GM IVD/由麻醉人員給予
  - 時間： 1420
  - 上次簽章: 吳婉寧

  ，追加預防性抗生素：
  - 抗生素名稱一： Veterin 1GM IVD由麻醉護理師執行
  - 時間： 1820
  - 上次簽章: 林愛甯
- 手術醫師： 蔣鋒帆，楊曙榮，古朋育，陳璿夫
- 麻醉醫師： VS 陳永銘
-
  - 刷手護士A ： 徐敬家，時間： 1340，刷手護士B ： 歐真勤，時間： 1340，接替刷手一： 盧陵怡，時間： 1500，接替刷手二： 吳思薇，時間： 1800
  - 流動護士A ： 吳婉寧，時間： 1340，接替流動一： 林愛甯/吳思薇，時間： 1500，接替流動二： 吳思薇/陳昱穎，時間： 1730
- 更多接替及流動護士： 接替流動三： 林愛甯/陳昱穎，時間： 1800，接替流動四： 林愛甯/許芷萱，時間： 1830

皮膚狀況

A-V Shunt： 無
術前： 完整
記錄者： 上次簽章: 吳婉寧
術後： 完整
記錄者： 林愛甯

手術準備

手術臥式 單一手術臥式

-
  - 手術臥式： 截石臥式
  - 使用附件： 溫毯 38 ℃，頭圈，腳架，其它： 海綿*2
  - 皮膚準備： Hibiscrub，2% Chlorhexidine
  - 電燒： 使用電燒: 電極片位置： 右大腿，雙極電燒，記錄者： 上次簽章: 吳婉寧

止血帶T： 無

植入物

- 植入物名稱/號碼/數量/部位：Covidien GIA 80mm*4.8mm/1/腸斷端
  Ethicon EEA 33mm/1/腸吻合處

，記錄者： 上次簽章: 吳思薇

標本送檢

- 細菌培養 無
- 細胞學 無
- 冰凍切片 無
- 病理標本

組織名稱一： vaginal wall
,
組織名稱二： IMA root
,
組織名稱三： distal cut end
, 更多組織名稱
  - 組織名稱四： rectum
- 截肢 無

標本交付 標本： 1 袋 ， 標本： 0 盒 ， 標本： 3 瓶 ， 細胞學： 0 管 ， Culture Tube： 0 管 ， 被交付醫師： R 陳瑋夫 ,

- 記錄者： 林愛甯
- 時間： 1910

傷口術後數料

傷口數料：　　　傷口數料： 無

- 請於圖上標示部位：

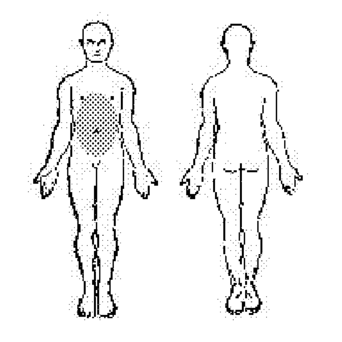

腹部trocar洞

- 傷口數料： mepore

導管

導管	天數	導管資訊	插入日期	插入時間	更換日期	拔除日期	拔除時間	拔除原因	預計拔除/更換	預計拔除/更換日期
A-line (1)	1	號碼:20 G; 部位:左手;	2021/06/10	1405						
Foley(1)	1	號碼:16 Fr.; 部位:膀胱;	2021/06/10	1400						
Epidural PCA(1)	1	部位:硬脊膜;	2021/06/10	1200						
Double Lumen CVC(1)	1	號碼:7 fr ; 深度:15CM; 部位:jugular (左); 象限:LU;	2021/06/10	1405						

用物計數

- 手術中出血量： minimal c.c.
- 手術中尿量： 150 c.c.
-
  - 其它： 無

用物計數： 多切口同時縫合

- 1.器械： 正確 , 2.縫針： 正確 , 3.刀片： 正確 , 4.紗布： 正確 , 5.腹部墊： 未使用 , 6.紗球： 未使用 , 7.PEANUTS ： 未使用 , 8.紗條： 未使用 , 9.絲帶： 未使用 , 11.Vessel loop： 未使用 , 12.Boots： 未使用 , 13.Jelco： 未使用 , 14.棉條： 未使用 ,
  - 第一次：
    - 刷手護士 ： 盧陵怡//陳昱穎
    - 流動護士： 上次簽章: 吳思薇
    - 時間： 1755

,
  - 第二次：
    - 刷手護士 ： 吳思薇/許芷萱
    - 流動護士： 上次簽章: 林愛甯

- 時間： 1835

,

- 第三次：
  - 刷手護士 ： 吳思薇
  - 流動護士： 上次簽章: 林愛甯
  - 時間： 1905

備註

備註：davinci 30度 XI scopy:No.2

- 記錄者： 上次簽章: 吳婉寧
- 時間： 1410

填寫護理人員 林愛甯

正式摘要

W95 / W95

doctorRank:B
